# Sinomenine exerts anticonvulsant profile and neuroprotective activity in pentylenetetrazole kindled rats: involvement of inhibition of NLRP1 inflammasome

**DOI:** 10.1186/s12974-018-1199-0

**Published:** 2018-05-18

**Authors:** Bo Gao, Yu Wu, Yuan-Jian Yang, Wei-Zu Li, Kun Dong, Jun Zhou, Yan-Yan Yin, Da-Ke Huang, Wen-Ning Wu

**Affiliations:** 1 Department of Pharmacology, School of Basic Medical Sciences, Key Laboratory of Anti-inflammatory and Immunopharmacology, Anhui Medical University, Hefei, 230032 People’s Republic of China; 2 Department of Psychiatry and Medical Experimental Center, Jiangxi Mental Hospital/Affiliated Mental Hospital of Nanchang University, Nanchang, 330029 People’s Republic of China; 3 Department of Pharmacy, Xi’an Chest Hospital, Shaanxi University of Chinese Medicine, Xi’an, 710061 People’s Republic of China; 4 Synthetic Laboratory, School of Basic Medical Sciences, Anhui Medical University, Hefei, 230032 People’s Republic of China

**Keywords:** Epilepsy, Inflammation, Sinomenine, Pentylenetetrazole, NLRP1 inflammasome, Neuroprotection

## Abstract

**Background:**

Epilepsy is a common neurological disorder and is not well controlled by available antiepileptic drugs (AEDs). Inflammation is considered to be a critical factor in the pathophysiology of epilepsy. Sinomenine (SN), a bioactive alkaloid with anti-inflammatory effect, exerts neuroprotective activity in many nervous system diseases. However, little is known about the effect of SN on epilepsy.

**Methods:**

The chronic epilepsy model was established by pentylenetetrazole (PTZ) kindling. Morris water maze (MWM) was used to test spatial learning and memory ability. H.E. staining and Hoechst 33258 staining were used to evaluate hippocampal neuronal damage. The expression of nucleotide oligomerization domain (NOD)-like receptor protein 1 (NLRP1) inflammasome complexes and the level of inflammatory cytokines were determined by western blot, quantitative real-time PCR and enzyme-linked immunosorbent assay (ELISA) kits.

**Results:**

SN (20, 40, and 80 mg/kg) dose-dependently disrupts the kindling acquisition process, which decreases the seizure scores and the incidence of fully kindling. SN also increases the latency of seizure and decreases the duration of seizure in fully kindled rats. In addition, different doses of SN block the hippocampal neuronal damage and minimize the impairment of spatial learning and memory in PTZ kindled rats. Finally, PTZ kindling increases the expression of NLRP1 inflammasome complexes and the levels of inflammatory cytokines IL-1β, IL-18, IL-6, and TNF-α, which are all attenuated by SN in a dose- dependent manner.

**Conclusions:**

SN exerts anticonvulsant and neuroprotective activity in PTZ kindling model of epilepsy. Disrupting the kindling acquisition, which inhibits NLRP1 inflammasome-mediated inflammatory process, might be involved in its effects.

**Electronic supplementary material:**

The online version of this article (10.1186/s12974-018-1199-0) contains supplementary material, which is available to authorized users.

## Background

Epilepsy is one of the most common neurological disorders characterized by recurrent epileptic seizures and cognitive and behavior impairment [1, 2]. In worldwide, approximately 50 million people are suffering from this disorder [3]. Despite numerously available antiepileptic drugs (AEDs) have been used to treat epilepsy, they are not totally efficacious for all epilepsy patients [4]. These compounds are mainly symptomatic and have little effect on the underlying pathology of this disorder [5, 6]. Besides, substantial side effects of currently available AEDs greatly reduce the quality of life of patients [7, 8]. Thus, understanding the molecular mechanisms of epileptogenesis and developing novel antiepileptic agents that modify the epileptic process are still urgently needed.

Accumulating evidences from clinical and experimental studies indicate that brain inflammation might be a cause or a consequence of epilepsy [3]. On the one hand, the expression of pro-inflammatory cytokines, such as IL-1β, IL-6, and TNF-α, is increased in brains of epileptic animals [9, 10]. Similarly, the level of these proinflammatory cytokines is also increased in serum or cerebrospinal fluid of patients with epilepsy [11, 12]. On the other hand, anti-inflammatory treatment also displays antiepileptic and neuroprotective effects [13]. As a critical platform regulating inflammatory responses, inflammasomes have attracted more and more attentions in various CNS disorders [14]. Inflammasomes are multi-protein complexes that consist of a cytosolic pattern-recognition receptor (a member of nucleotide oligomerization domain (NOD)-like receptor (NLR) family or HIN domain-containing (PYHIN) family), an adaptor known as apoptosis-associated speck-like protein containing a caspase-activating recruitment domain (ASC) and pro-caspase-1 [15]. Various stimuli can trigger inflammasome assembly, and then cleave pro-caspase-1 into active capsase-1 resulting in the maturation of proinflammatory cytokines IL-1β and IL-18 [16–18]. Active IL-1β could stimulate the secretion of other cytokines including TNF-α and IL-6 [19, 20]. To date, many inflammasomes have been well characterized, such as NLRP1 (NLR protein 1), NLRP2, NLRP3, NLRC4 (CARD domain-containing protein 4) inflammasome, and AIM2 (absent in melanoma 2) inflammasome [21–25]. The NLRP1 inflammasome is the first to be characterized and expressed in neurons and glial cells [21, 26]. Recent study shows that NLRP1 inflammasome contributes to seizure-induced degenerative process in patients and in the animals with temporal lobe epilepsy (TLE) [27]. These indicate that NLRP1 inflammasome-mediated inflammatory processes might be a critical mediator in the physiopathology of epilepsy.

Sinomenine (SN), a bioactive alkaloid extracted from the Chinese medicinal plant *Sinomenium acutum*, has been used for the clinical treatment of rheumatoid arthritis in China [28]. Previous studies show that SN exhibits a variety of pharmacological effects, including anti-inflammation, immunosuppression, anti-tumor, and neuroprotection [29–31]. Recent studies indicate that SN exerts neuroprotective effect by inhibiting inflammatory processes [32–34]. However, the effect of SN on epilepsy, an inflammation-related neurological disorders, remains little known. In present study, anticonvulsant and neuroprotective effects of SN were investigated in PTZ kindling model of epilepsy. To determine SN’s related mechanism of action, we also examined the effects of the drug on NLRP1 inflammasome activation and the associated inflammatory processes.

## Methods

### Animals

Male Sprague-Dawley rats (250–300 g) were obtained from the Experimental Animal Center of Anhui Medical University. They were kept in a controlled environment with a temperature of 22 ± 2 °C and humidity of 60% under a 12 h light/dark cycle. Food and water were available ad libitum. All animal procedures were approved by the Committee for Experimental Animal Use and Care of Anhui Medical University.

### Chemicals

SN was purchased from Aladdin Industrial Corporation (Aladdin, Shanghai, China). PTZ and Hoechst 33258 were obtained from Sigma-Aldrich (St. Louis, MO, USA). Primary antibodies of Bax and Bcl-2 were purchased from Cell Signaling Technology Inc. (Danvers, MA, USA). Primary antibodies of NLRP1, caspase-1, IL-1β, IL-6, and TNF-α were purchased from Abcam (San Francisco, CA, USA). Primary antibodies of caspase-3, ASC, and IL-18 were purchased from Santa Cruz Biotechnology (Santa Cruz, CA, USA). Horseradish peroxidase-conjugated secondary antibodies were purchased from Santa Cruz Biotechnology (Santa Cruz, CA, USA). Other general agents were commercially available.

### Kindling procedure

The PTZ kindling epilepsy model was induced as previously described [35]. Briefly, rats were intraperitoneally (i.p.) injected with a sub-convulsive dose of PTZ (35 mg/kg) once every other day for 15 injections (29 days). Rats with three consecutive seizures of stage 4 were considered to be fully kindled [36]. Animals were observed for 30 min after each injection. The seizure intensity was scored as follows [36, 37]: stage 0, no response; stage 1, facial movements, ear, and whisker twitching; stage 2, myoclonic convulsions without rearing; stage 3, myoclonic convulsions with rearing; stage 4, clonic-tonic convulsions; stage 5, generalized clonic-tonic seizures with loss of postural control; stage 6, death. To investigate anticonvulsant and neuroprotective effects of SN, rats were divided into four groups as follow (Table 1): control group that received saline once every day. SN group that received SN (80 mg/kg, i.p.) once every day. PTZ group that received PTZ (35 mg/kg, i.p.) once every other day. PTZ + SN group that received different doses of SN (20, 40, and 80 mg/kg, i.p.) at 30 min prior to PTZ once every day. To further confirm anticonvulsant effect of SN, another experiment was performed to wash out SN. Rats were divided into four groups as follows (Table 2): control group that received saline once every day. PTZ group that received PTZ (35 mg/kg, i.p.) once every other day. PTZ + SN group that received SN (40 mg/kg, i.p.) at 30 min prior to PTZ once every day. SN washout (SNW) group that received SN (40 mg/kg, i.p.) at 30 min prior to PTZ once every day in the beginning. After 14 injections of PTZ, rats in SNW group were not received SN again and were only received PTZ until they were fully kindled.

**Table 1:** Experimental group for performing SN’s anticonvulsant and neuroprotective effects. PTZ was administered to rats every other day. Saline and SN were administered to rats at 30 min prior to PTZ once every day

Experimental group	Saline	PTZ	SN
Control	Yes	No	No
SN	No	No	80 mg/kg
PTZ	No	35 mg/kg	No
PTZ + SN	No	35 mg/kg	20 mg/kg
	No	35 mg/kg	40 mg/kg
	No	35 mg/kg	80 mg/kg

**Table 2:** Experimental group for performing SN washout. PTZ was administered to rats every other day. Saline and SN were administered to rats at 30 min prior to PTZ once every day. Rats in SNW (SN washout) group was only received PTZ after 14 PTZ injections

Experimental group	Number of PTZ injection (1–14)			Number of PTZ injection (15–19)		
	Saline	PTZ	SN	Saline	PTZ	SN
Control	Yes	No	No	Yes	No	No
PTZ	No	35 mg/kg	No	No	35 mg/kg	No
PTZ + SN	No	35 mg/kg	40 mg/kg	No	35 mg/kg	40 mg/kg
SNW	No	35 mg/kg	40 mg/kg	No	35 mg/kg	No

### Morris water maze test

Morris water maze (MWM) is mainly consisted of a black circular pool (diameter 160 cm, height 60 cm) filled with water (depth 30 cm, temperature 25 ± 2 °C) and an circular platform (diameter 10 cm) where animals can escape. The pool was divided into four equal quadrants, and escape platform was placed in a constant quadrant (target quadrant) and was submerged 1.5 cm below the water surface. Several distal extra-maze cues, which used for spatial orientation, were placed around the pool and remained in the same position throughout experiment. MWM test was performed at the end of kindling procedure. Only 1 day prior to the first training trial, animals were allowed an adaptation period (swim freely for 120 s with no platform present) in the pool. For acquisition trial, rats underwent four trials per day with a 30 min intertrial interval for five consecutive days. In each trial, rats were placed into the water starting from one of four quadrants with its head facing towards the wall of the pool. Each rat was allowed to swim until finding the platform. Maximal duration of each trial is 120 s. After climbing on the platform, the rat was allowed to remain there for 15 s, and then was removed and released from the next starting point. The escape latency and the swimming track were recorded. If the rat failed to find the hidden platform within 120 s, it was guided to platform manually and was allowed to stay there for 15 s. Its escape latency was recorded as 120 s. For the spatial probe test, the hidden platform was removed on the sixth day. The rat was released from quadrant which was opposite to the target quadrant and was allowed to swim freely for 120 s. The times of crossing the former platform area and the time spent in target quadrant were recorded. After the probe trial, visible platform test was performed to evaluated sensorimotor ability and motivation. For this test, the escape platform was raised 2 cm above the water level. The escape latency and swimming speed were recorded. After each swimming session, animals were allowed to warm up and dry off before they were returned to the home cage.

### Histological assay and Hoechst 33258 staining

24 h after last injection of PTZ, rats were anesthetized and the brains were removed quickly and fixed in 4% paraformaldehyde. Paraffinized brains were cut into 5 μm sections using microtome and were stained with hematoxylin and eosin (H.E.). The morphology of hippocampal CA1 and CA3 areas was examined by light microscope (Olympus IX71, Tokyo, Japan). For Hoechst 33258 staining, paraffin sections described above were deparaffinized with xylene twice and then washed with PBS for five times. After incubated with Hoechst 33258 (25 mM) for 15 min, the sections were washed with PBS for three times and mounted onto slides. The cells showing nuclear condensation under fluorescence microscopy (Olympus IX71, Tokyo, Japan) were counted for evaluating neuronal apoptosis.

### Western blotting

24 h after last injection of PTZ, animals were sacrificed by decapitation and hippocampus was isolated. Dissected hippocampal tissues were homogenized in lysis buffer containing 50 mM Tris-base (pH 7.4), 100 mM NaCl, 1% NP-40, 10 mM EDTA, 20 mM NaF, 1 mM PMSF, and protease inhibitors. After being lysed for 30 min on ice, samples were centrifuged at 12,000 *g* at 4 °C for 15 min. Supernatant was separated, and protein concentration was determined using the BCA protein assay kit (Pierce Biotechnology, Inc., Rockford, IL, USA). Protein samples (30 μg) were separated by 10% SDS-polyacrylamide gel and then transferred onto a polyvinylidencefluoride membrane (Millipore). After blocking with 5% nonfat milk in Tris-buffered saline containing 0.1% Tween-20 (TBST) for 1 h at room temperature, membranes were incubated overnight at 4 °C with different primary antibodies (anti-NLRP1, anti- caspase-1, anti-IL-1β, anti-IL-6 and anti-TNF-α, 1:800 dilution; anti-Bax and anti-Bcl2, 1:500 dilution; anti-caspase-3, anti-ASC, and anti-IL-18, 1:200 dilution) followed by incubation with horseradish peroxidase-conjugated secondary antibodies (1:10000 dilution) in TBST with 1% nonfat milk for 1 h at room temperature. And then membranes were reacted with enhanced chemiluminescence reagents (Amersham Pharmacia Biotech, Inc., Piscataway, NJ, USA) for 5 min and were visualized using chemiluminescence detection system (Bioshine, Shanghai, China).

### Quantitative real-time PCR analysis

Hippocampal tissues were dissected as described above. Total RNA was extracted from hippocampus using TRIzol reagent (Invitrogen, USA) following the manufacturer’s instructions. cDNA synthesis was performed using a PrimeScript first Strand cDNA Synthesis Kit (Takara Biotechnology). PCR amplifications of cDNA were performed by standard methods. The following specific primers were used: NLRP1 (forward: 5-GCCCTGGAGACAAAGAATCC-3, reverse: 5-AGTGGGCATCGTCATGTGT-3); ASC (forward: 5-ACCCCATAGACCTCACTG AT-3, reverse: 5-ACAGCTCCAGACTCTTCCAT-3); Caspase-1 (forward: 5-ATGCC GTGGAGAGAAACAAG-3, reverse: 5-CCAGGACACATTATCTGGTG-3); β-actin (forward: 5-TTCCTTCCTGGGTATGGAAT-3, reverse: 5-GAGGAGCAATGATCTT GATC-3). The fluorescent signals were collected during extension stage, Ct values of the sample were calculated and relative transcript levels were analyzed by 2^−ΔΔCt^ method.

### Enzyme-linked immunosorbent assay (ELISA)

24 h after last injection of PTZ, rats were sacrificed by decapitation and hippocampal tissues were dissected. The protein samples were extracted and protein concentration was determined as described above. The levels of inflammatory cytokines IL-1β, IL-18, IL-6, and TNF-α in hippocampus were measured by commercial ELISA kits (R&D Systems, Minneapolis, MN, USA) according to the manufacturer’s protocol.

### Statistical analysis

All data were analyzed by analysis of variance (ANOVA) with the statistical program SPSS 17.0 (Chicago, IL, USA). Data related to seizure stage and escape latency in MWM test were analyzed using two-away ANOVA with repeated measures followed by Bonferroni or Dunnett’s T3 post hoc test. Other data were analyzed by one-away ANOVA. Data are expressed as means ± SEM. *P* < 0.05 was considered statistically significant.

## Results

### SN exerts anticonvulsant profile in PTZ kindling model of epilepsy

**Fig. 1:**
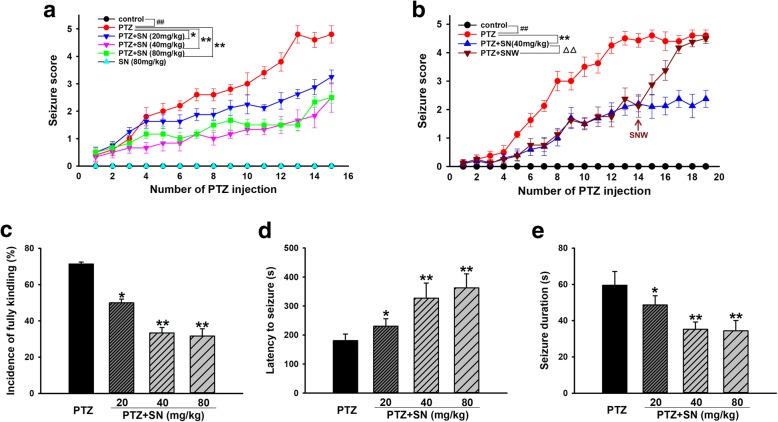
Effects of SN on PTZ kindling-induced seizure. **a** Statistical results showing SN decreased seizure score in a dose-dependent manner. **b** Statistical results showing seizure score is increased after SN is washed out. **c** Statistical results showing SN decreased the incidence of fully kindling. **d** Statistical results showing SN increased the latency to generalized seizures. **e** Statistical results showing SN decreased the duration of generalized seizures. Data are expressed as means ± SEM. *n* = 12–15, ^##^*P* < 0.01 vs control and *P < 0.05 and **P < 0.01 vs PTZ

Firstly, anticonvulsant effect of SN was investigated. Seizure stage scores and fully kindled incidence were recorded. We also recorded the latency (the duration from PTZ administration to seizure event) and duration of generalized seizures (stage 4 or greater). As shown in Fig. 1a, Seizure stage in PTZ group reached 4.40 ± 0.22 after 15 injections. SN alone did not influence the behavior of rats, but 20, 40, and 80 mg/kg SN treatment reduced seizure stage to 3.25 ± 0.15, 2.50 ± 0.74, and 2.5 ± 0.20, respectively. However, SN washout reversed its effect on seizure stage (Fig. 1b), indicating that SN disrupted the kindling acquisition processes. Moreover, 20, 40, and 80 mg/kg SN treatment decreased the incidence of fully kindling from 71.43 to 50, 33.33, and 31.67% in PTZ kindled rats, respectively (Fig. 1c). In addition, SN also significantly increased the latency to generalized seizures and reduced the duration of generalized seizures in a dose-dependent manner (Fig. 1d, e), indicating that SN exhibits anticonvulsant activity in PTZ kindled rats.

### SN minimizes kindling-induced spatial learning and memory deficits in rats

**Fig. 2:**
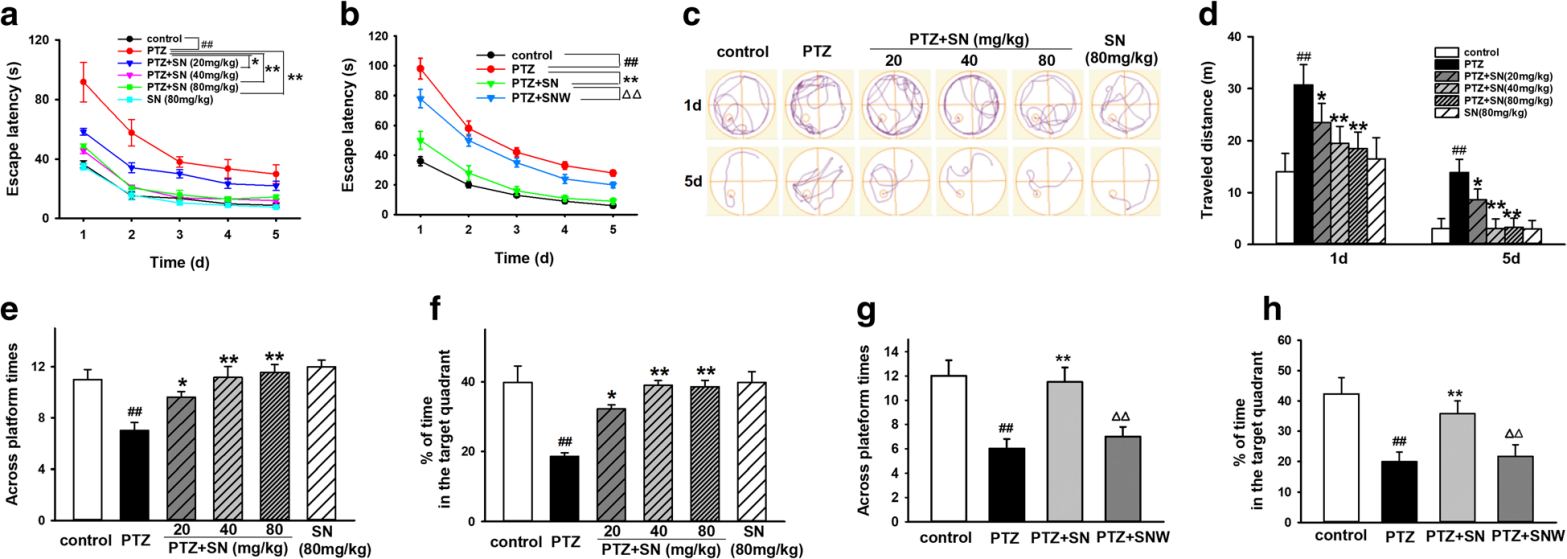
Effects of SN on cognitive deficits induced by PTZ kindling. **a** Statistical results showing SN decreased escape latency. **b** Statistical results showing SN washout reversed its effect on escape latency. **c**, **d** Representative traces and statistical results showing rat’s swimming distance searching for hidden platform in the first and fifth training day. **e**, **f** Statistical results showing SN dose-dependently increased the number of times crossing the target quadrant and the time spent in the target quadrant in PTZ kindling rats. **g**, **h** Statistical results showing SN washout reversed its effect on the number of times crossing the target quadrant and the time spent in the target quadrant. Data are expressed as means ± SEM. *n* = 10–12, ^##^*P* < 0.01 vs control and *P < 0.05 and **P < 0.01 vs PTZ and ^△△^*P* < 0.01 vs PTZ + SN

Then, we performed MWM test to assess the effect of SN on spatial learning and memory of PTZ kindling rats. In acquisition trial, the escape latency of all groups decreased gradually during five training days. The escape latency of rats of PTZ group was significant longer than that of control group. There were no statistical differences between SN alone group and control group. However, SN dose-dependently reduced the escape latency (Fig. 2a). While SN washout reversed its effect on escape latency (Fig. 2b). In the fifth training day, rats in PTZ group swam a longer distance to reach the hidden platform compared with control group. Treatment with different doses of SN significantly decreased the swimming distance to find the hidden platform (Fig. 2c, d). In probe trail, rats in PTZ group showed a decrease in the number of times crossing the target quadrant and the time spent in the target quadrant compared with control group. There were no statistical differences between SN alone group and control group. However, SN treatment significantly increased the number of times crossing the target quadrant and the time spent in the target quadrant in a dose-dependent manner (Fig. 2e, f). While SN washout reversed its effect on the number of times crossing the target quadrant and the time spent in the target quadrant (Fig. 2g, h). To rule out the effect of sensorimotor ability and motivation on the results, we performed visible platform test and found there were no statistical differences in the escape latency and swimming speed among all groups (Additional file 1), indicating that the alteration of all parameters above did not result from the sensorimotor ability of rats. All these data suggested that SN minimizes the impairment of spatial learning and memory induced by PTZ kindling in rats.

### SN blocks hippocampal neuronal damage in PTZ kindled rats

**Fig. 3:**
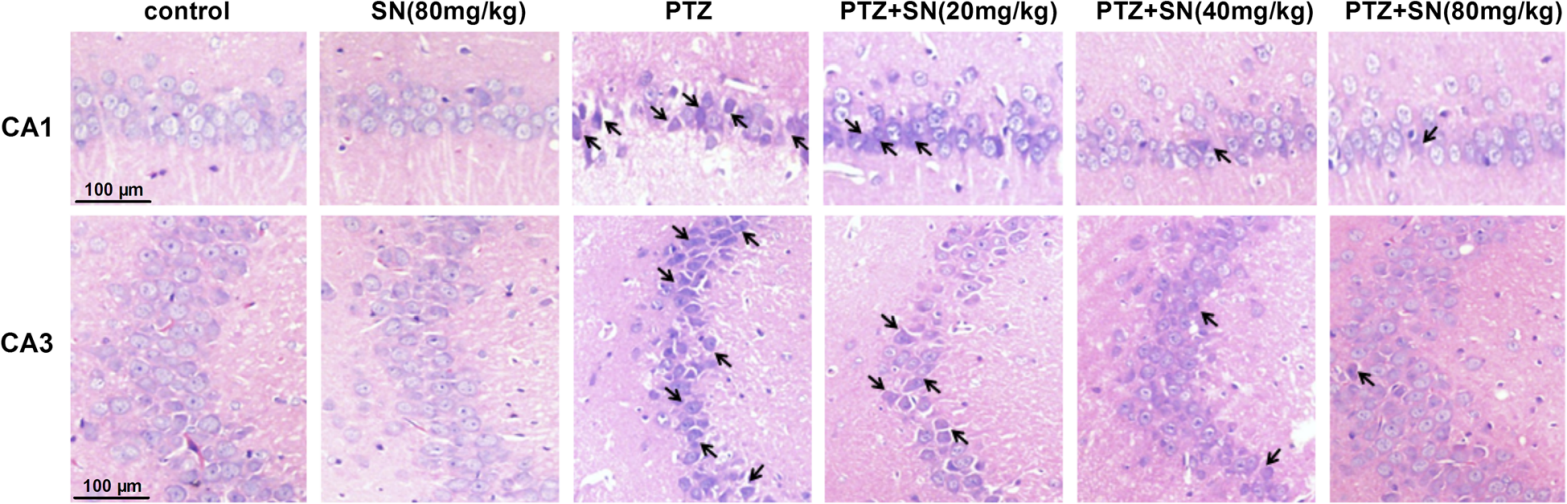
Effects of SN on hippocampal neuronal damage induced by PTZ kindling. Representative micrographs (original magnification, × 200) showing SN blocked hippocampal neuronal damage (arrow) induced by PTZ kindling in CA1 and CA3 areas in a dose-dependent manner. Scale bar = 100 μm

**Fig. 4:**
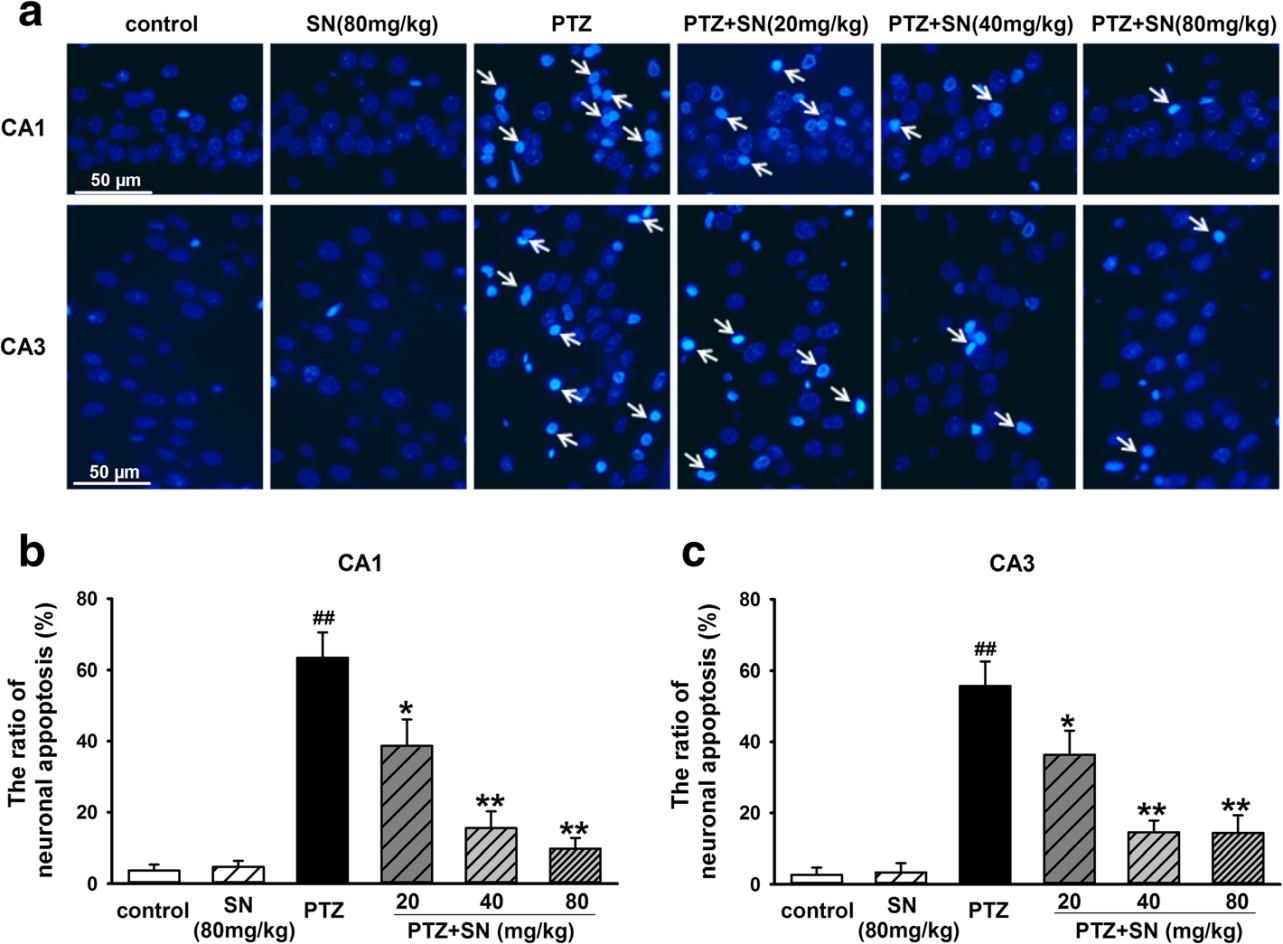
Effects of SN on hippocampal neuronal apoptosis induced by PTZ kindling. **a** Representative imagines (original magnification, × 400) showing SN inhibited hippocampal neuronal apoptosis (arrow) induced by PTZ kindling in CA1 and CA3 areas in a dose-dependent manner. Scale bar = 50 μm. Statistical results showing different doses of SN treatment reduced the number of apoptotic neuron in hippocampal CA1 (**b**) and CA3 (**c**) areas. Data are expressed as means ± SEM. *n* = 8–10, ^##^*P* < 0.01 vs control and *P < 0.05 and **P < 0.01 vs PTZ

**Fig. 5:**
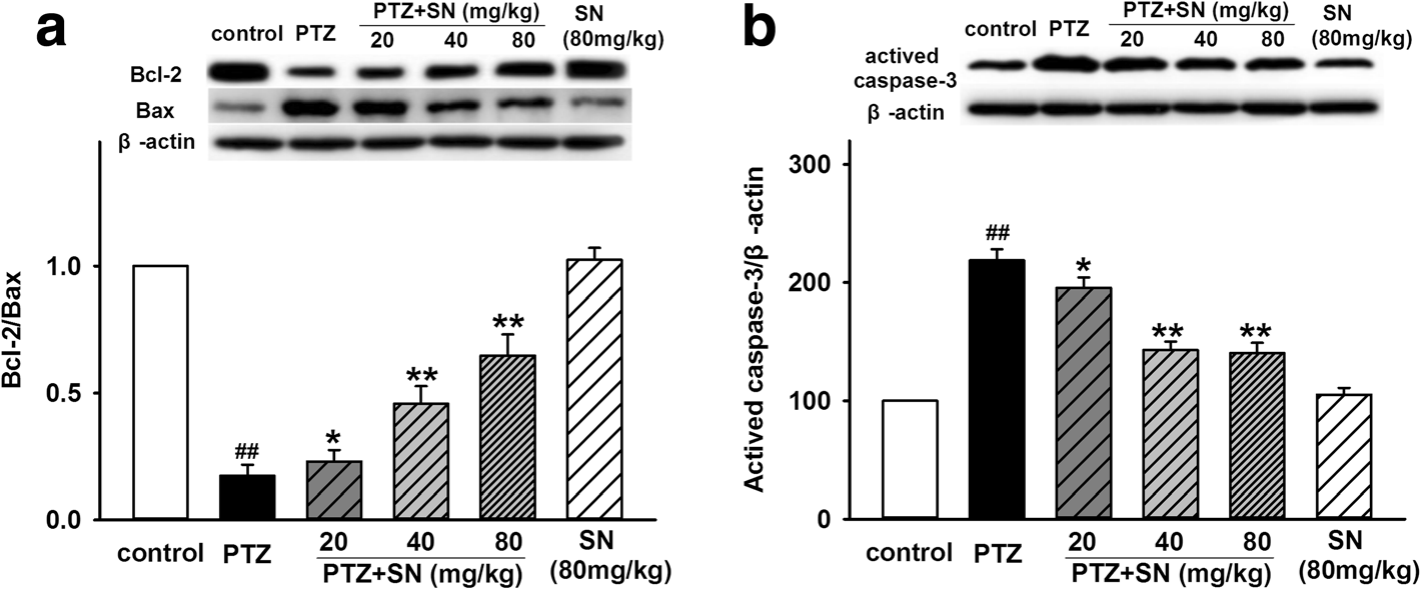
Effects of SN on hippocampal apoptosis-related proteins in PTZ kindled rats. **a** Representative immunoreactive bands and statistical results showing SN prevented PTZ-induced decrease in the ratio of Bcl-2/Bax in a dose-dependent manner. **b** Representative immunoreactive bands and statistical results showing SN prevented PTZ-induced increase in the expression of activated caspase-3 in a dose-dependent manner. Data are expressed as means ± SEM. *n* = 6, ^##^*P* < 0.01 vs control and *P < 0.05 and **P < 0.01 vs PTZ

Hippocampus has long been known as a critical structure for spatial learning and memory [38], so we subsequently investigated the effects of SN on hippocampal neuronal damage induced by PTZ kindling. First, histological examination was performed by H.E. staining. As shown in Fig. 3, hippocampal CA1 and CA3 areas of rats in PTZ group exhibited a serious damage compared with control group. SN dose-dependently blocked hippocampal neuronal damage induced by PTZ kindling. Then, neuronal apoptosis was evaluated by Hoechst 33258 staining. Compared with control group, the number of apoptotic neurons in hippocampal CA1 and CA3 areas was significantly increased in PTZ group. There were no statistical differences between SN alone group and control group. However, SN dose-dependently prevented neuronal apoptosis induced by PTZ kindling (Fig. 4). Finally, apoptosis-related proteins caspase-3, Bax, and Bcl2 in the hippocampus were detected by western blot. Compared with control group, the expression of caspase-3 and Bax was significantly increased, while the expression of Bcl-2 was decreased in PTZ group. The ratio of Bcl-2/Bax was also decreased in PTZ group. There were no statistical differences between SN alone group and control group. However, the effects of PTZ kindling could be inhibited by SN in a dose-dependent manner (Fig. 5). While SN washout reversed its effect on the expression of caspase-3, Bax, and Bcl2 (Additional file 2). All these results suggest that SN blocks hippocampal neuronal damage and apoptosis from PTZ kindling.

### SN inhibits NLRP1 inflammasome activation in PTZ kindled rats

**Fig. 6:**
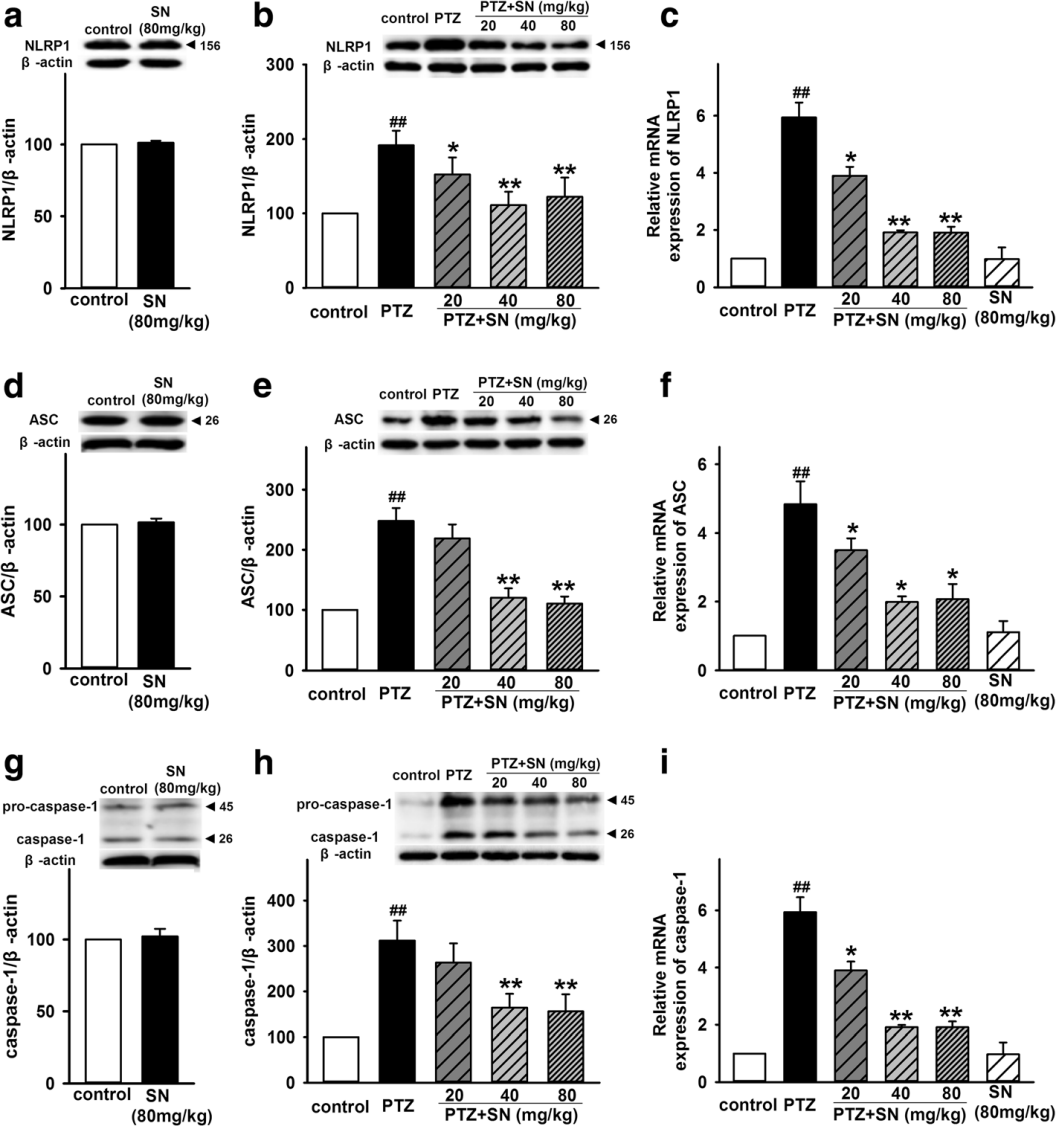
Effects of SN on the expression of hippocampal NLRP1 inflammasome complexes in PTZ kindled rats. **a**, **d**, and **g** Representative immunoreactive bands and statistical results showing SN alone has no influence on the protein expression of NLRP1, ASC, and caspase-1 in protein level. **b**, **e,** and **h** Representative immunoreactive bands and statistical results showing SN dose-dependently inhibited PTZ-induced increase in the protein expression of NLRP1, ASC, and caspase-1 in protein level. **c**, **f,** and **i** Statistical results showing SN dose-dependently inhibited PTZ-induced increase in the expression of NLRP1, ASC, and caspase-1 at mRNA level. Data are expressed as means ± SEM. *n* = 6–8, ^##^*P* < 0.01 vs control and *P < 0.05 and **P < 0.01 vs PTZ

Previous study has demonstrated that NLRP1 inflammasome was activated in TLE patients and electrical kindling model [27]. In order to determine the effect of PTZ kindling on NLRP1 inflammasome activation, NLRP1 inflammasome complexes in hippocampus was detected at protein and mRNA level. As shown in Fig. 6b, the expression of NLRP1 protein of rats in PTZ group is significantly increased compared with control group. Treatment with different doses of SN significant inhibited the effect, while SN alone did not influence NLRP1 expression (Fig. 6a). Similarly, higher doses of SN (40 and 80 mg/kg) also prevented the increase of ASC and caspase-1 expression induced by PTZ kindling (Fig. 6d, e, g, and h) while SN washout reversed its effect on the expression of NLRP1, ASC, and caspase-1 (Additional file 3). Our results also showed that the levels of NLRP1 mRNA, ASC mRNA, and caspase-1 mRNA of rats in PTZ group were significantly higher than those of rats in control group. The effects were significantly inhibited by SN in a dose-dependent manner, while SN alone did not influence the expression of NLRP1, ASC, and caspase-1 at mRNA level (Fig. 6c, f, i). These data suggest that NLRP1 inflammasome activation is associated with acquisition of the fully kindled state in PTZ kindling model and that SN disrupts kindling acquisition which contributes to the inhibition of NLRP1 inflammasome activation.

### SN decreases the levels of inflammatory cytokines in hippocampus of PTZ kindled rats

**Fig. 7:**
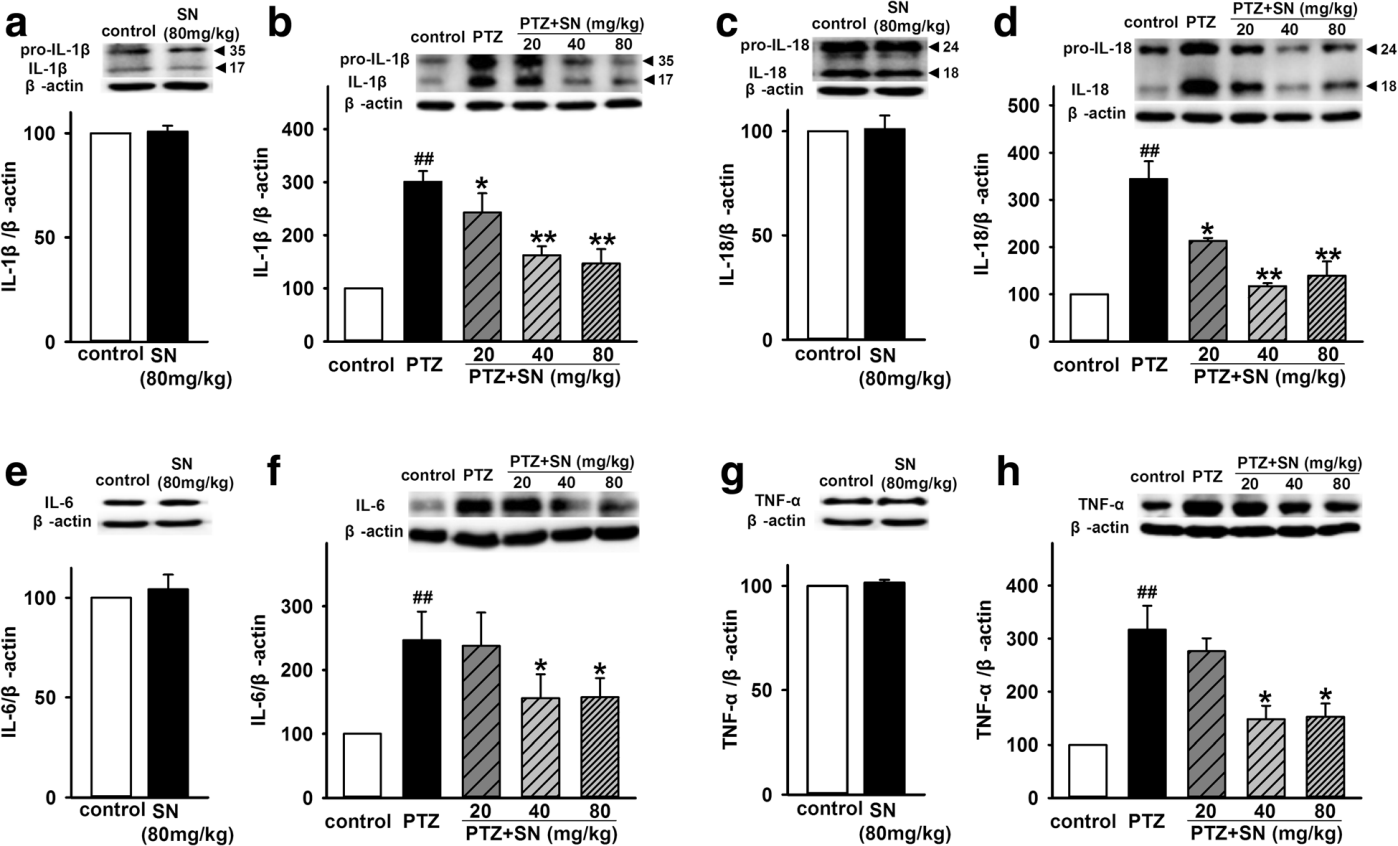
Effects of SN on the expression of hippocampal inflammatory cytokines in PTZ kindled rats. Representative immunoreactive bands and statistical results showing SN alone has no influence on the expression of IL-1β (**a**), IL-18 (**c**), IL-6 (**e**), and TNF-α (**g**) in protein level. Representative immunoreactive bands and statistical results showing SN dose-dependently inhibited PTZ-induced increase in the expression of IL-1β (**b**), IL-18 (**d**), IL-6 (**f**), and TNF-α (**h**) in protein level. Data are expressed as means ± SEM. *n* = 6–8, ^##^*P* < 0.01 vs control and *P < 0.05 and **P < 0.01 vs PTZ

**Fig. 8:**
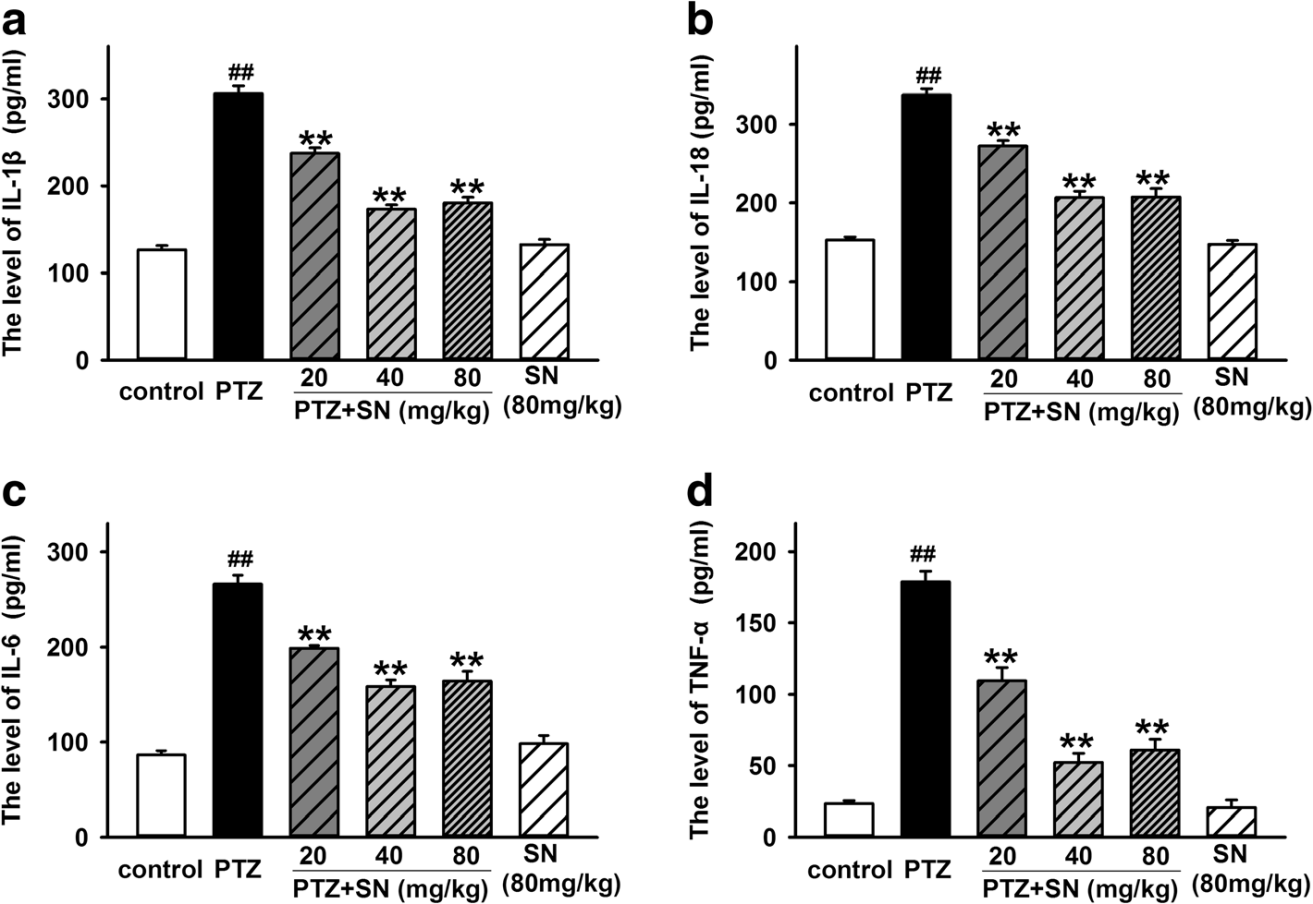
Effects of SN on the content of inflammatory cytokines in hippocampus homogenate in PTZ kindled rats. Statistical results showing SN dose-dependently inhibited PTZ-induced increase in the content of IL-1β (**a**), IL-18 (**b**), IL-6 (**c**), and TNF-α (**d**). Data are expressed as means ± SEM. *n* = 8, ^##^*P* < 0.01 vs control and **P < 0.01 vs PTZ

As a key regulator of innate immune and inflammatory response, inflammasome directly or indirectly promotes the secretion of inflammatory cytokines, such as IL-1β, IL-18, IL-6, and TNF-α. To further determine the effect of SN on pro-inflammatory cytokines in PTZ kindled rats, we first detected the levels of IL-1β, IL-18, IL-6, and TNF-α in hippocampus by western blot. As shown in Fig. 7b, d, the expression of IL-1β and IL-18 in PTZ group was significant increased compared with control group. The effects were significantly attenuated by SN in a dose-dependent manner, while SN alone did not influence their expression (Fig. 7a, c). Similarly, higher doses of SN (40 and 80 mg/kg) also prevented the increase of IL-6 and TNF-α expression induced by PTZ kindling (Fig. 7e, f, g, h) while SN washout reversed its effect on the expression of IL-1β, IL-18, IL-6, and TNF-α (Additional file 4). Moreover, we also detected the levels of IL-1β, IL-18, IL-6, and TNF-α in hippocampus by ELISA kits. Compared with control group, the levels of IL-1β, IL-18, IL-6, and TNF-α were significantly increased in PTZ group. There were no statistical differences between SN alone group and control group. However, the effects of PTZ were significantly inhibited by SN in a dose-dependent manner (Fig. 8), which is consistent with western blot results. Together, these results indicate that SN inhibits NLRP1 inflammasome-mediated inflammatory processes in PTZ kindled rats.

## Discussion

In the current study, we demonstrated that SN delayed kindling acquisition and decreased the severity of seizure in PTZ kindled rats. Also, we found that SN blocked hippocampal neuronal damage and cognitive deficits. In addition, our results also showed that SN inhibited NLRP1 inflammasome activation and reduced the secretion of inflammatory cytokines. Interestingly, SN washout blocked these effects, suggesting that disrupting kindling acquisition may be responsible for the anticonvulsant and neuroprotective effects of SN.

Epilepsy is recognized as a complex clinical syndrome. Excessively excitation of CNS resulting from imbalance between excitation and inhibition is considered as the primary cause of epilepsy [39]. However, pathogenesis of epilepsy is still not well understood, and consequently, approximate 30% of patients suffering from seizure episodes under treatment with AEDs [40, 41]. Increasing evidence has shown that inflammatory processes within brain may be a crucial mechanism in the pathophysiology of epilepsy and inflammation is considered as a biomarker of epileptogenesis [42, 43]. Inflammatory mediators can trigger neuronal hyperexcitability by activating specific signaling such as NMDA receptor and Toll-like receptor 4, and then result in increased probability of seizure recurrence. Anti-inflammatory treatments can drastically reduce spontaneous seizure frequency and the severity of the disease [13]. In addition, cyclooxy-genase-2 (COX-2) is expressed at low level in hippocampal neurons, but it is markedly increase within an hour after a seizure [44], and diclofenac sodium, a COX-inhibitor, has been reported to reduce the severity of seizure in PTZ kindling model [45].

In view of the above mentioned reasons, we speculated that SN with anti-inflammatory effect, considered as an inhibitor of COX-2 [46], may be able to protect against seizure. To test this hypothesis, a chronic epilepsy model was established by PTZ kindling which could simulate clinical seizure and was widely accepted as an experimental animal model for epileptogenesis. Our results showed that most rats were fully kindled after a subconvulsive dose of PTZ (35 mg/kg, i.p.) was administrated once every other day for 15 injections (29 days). As we expected, SN alone did not influence animal’s behavior. However, SN could dose-dependently decrease the severity and incidence of fully kindled seizure, while SN washout increased the severity of seizure (Fig. 1a–c), indicating that SN delayed the kindling acquisition process. Furthermore, SN also significantly increased the latency and reduced the duration of generalized seizures (Fig. 1d, e). These indicate that SN disrupts the kindling acquisition and exerts an anticonvulsant effect in PTZ-induced seizure rats.

As we know, cognitive deficits usually accompany epilepsy. Learning and memory impairments have been found in patients with TLE and electrical kindling model, as well as chemical kindling model induced by PTZ and kainic acid (KA) [47–49]. Although several treatment strategies and AEDs are applied, they exhibit little effectiveness in cognitive deficits even under controlled seizure [50]. Therefore, after determining the anticonvulsant effect of SN, we investigated the effect of SN on cognitive deficits in PTZ kindled rats by MWM test. Our results showed that the escape latency of PTZ kindled rats was still longer compared with control group, indicating PTZ impaired spatial learning ability of rats, which is consistent with previous reports. SN dose-dependently decreased the escape latency and improved spatial learning ability of rats (Fig. 2a), while SN washout increased the escape latency (Fig. 2b). Also, after the hidden platform was removed, the number of times crossing the target quadrant and the time spent in the target quadrant were decreased in PTZ kindled rats. And the effect could be inhibited by SN in a dose-dependent manner, suggesting that SN could alleviate the impairment of spatial memory induced by PTZ kindling (Fig. 2e, f), while SN washout reversed its effect on spatial learning ability (Fig. 2g, h). In addition, we performed visible platform test to exclude vision and motor interference in behavior test. The results showed that there were no statistical differences in escape latency and swimming speed among all groups (Additional file 1). These data suggest that SN can prevent the impairment of spatial learning and memory from PTZ kindling.

Hippocampus has long been known to be crucial for learning and memory in mammals [51]. Neuronal damage and dysfunction in this area will result in cognitive deficits [52]. Previous studies have shown that PTZ kindled seizure leads to hippocampal neuronal damage followed by spatial learning and memory impairment [35, 53]. Our data showed that SN protected against PTZ-induced seizure and improved the impairment of spatial learning and memory. SN also exhibited neuoprotection in vitro and in vivo [31, 34]. Thus, we proposed that SN might protect against hippocampal neuronal damage induced by PTZ kindling. To test this hypothesis, we first performed a series of experiments to evaluate the effect of SN on hippocampal neuronal damage in PTZ kindled rats. Our results showed SN can dose-dependently block neuronal damage (Fig. 3) and inhibit neuronal apoptosis in the hippocampal CA1 and CA3 areas of PTZ kindled rats (Fig. 4). Moreover, SN inhibited the expression of pro-apoptosis protein Bax and caspase-3 and increased the expression of anti-apoptosis protein Bcl-2 in a dose-dependent manner (Fig. 5), while SN washout reversed its effect on apoptosis-related proteins (Additional file 2). All these suggest that SN can block hippocampal damage induced by PTZ kindling and exhibits a neuroprotective effect, which may contribute to its improvement on behavior and cognitive deficits.

Inflammation is a homeostatic mechanism of defense against noxious stimuli and is designed to limit harm to the host. Neuroinflammation has been found in the pathological processes of many CNS diseases such as autoinmmune, neurodegenerative, epileptic, and psychiatric disorders [54]. Inflammasomes are multi-protein complexes discovered in 2002 [21] and has been known to be responsible for activation of inflammatory processes resulting in the secretion of inflammatory cytokines such as IL-1β, IL-18, IL-6, and TNF-α [55]. Inflammasomes-mediated inflammatory pathway has involved in various CNS disorders and leads to neuronal damage and changes in behavior [14]. As the first identified inflammasome, NLRP1 inflammasome has been reported to involve in the pathological processes of many nervous system diseases such as spinal cord injury (SCI), traumatic brain injury (TBI), Alzheimer’s disease (AD), and nociception [56]. Recent study showed that the level of NLRP1 and caspase-1 is upregulated in TEL patients and electrical kindling model, and NLRP1 or caspase-1 silencing exhibited an antiepileptic and neuroprotective effects [27], indicating NLRP1 inflammasome play a critical role in seizure-induced neuronal damage. Here, we have demonstrated that SN disrupted the kindling acquisition and exerted antiseizure and neuroprotective effects. To further investigate the neuroprotective effects of SN, we investigated the effect of SN on NLRP1 inflammasome activation and associated inflammatory processes in PTZ kindled rats. Similar to previous report, our results showed that PTZ kindling also increased the expression of NLRP1, ASC, and caspase-1 at protein and mRNA levels. And SN can inhibit the effects of PTZ in a dose-dependent manner (Fig. 6). Furthermore, we found that SN also inhibited the upregulation of pro- inflammatory cytokines IL-1β, IL-18, IL-6, and TNF-α in PTZ kindled rats (Figs. 7 and 8), while SN washout reversed its effect on NLRP1 inflammasome activation and inflammatory response (Additional file 3 and 4). These data indicate that disrupting kindling acquisition may contribute to inhibitory effect of SN on NLRP1 inflammasome-mediated inflammatory processes, which may be involved in neuroprotective effects of SN. However, the precise mechanism that SN regulates NLRP1 inflammasome signal is unclear. Further efforts will be made to clarify it in future research.

## Conclusions

The present study showed that SN exerts anticonvulsant profile and neuroprotective effects in PTZ kindling model of epilepsy. These effects may be associated with disrupting kindling acquisition resulting in inhibition of NLRP1 inflammasome-mediated inflammatory processes.

## Additional files

Additional file 1: **Figure S1.** The effect of sensorimotor ability and motivation on the escape latency and swimming speed. (A) and (B) Statistical results showing there were no differences in the escape latency and swimming speed among all groups in visible platform test. Data are expressed as means ± SEM. *n* = 10–12, *P* > 0.05. (PDF 71 kb)

Additional file 2: **Figure S2.** SN washout reverses its effect on hippocampal apoptosis-related proteins in PTZ kindled rats. (A) Representative immunoreactive bands and statistical results showing SN washout reversed its effect on the ratio of Bcl-2/Bax. (B) Representative immunoreactive bands and statistical results showing SN washout reversed its effect on the expression of activated caspase-3. Data are expressed as means ± SEM. *n* = 6, ^##^*P* < 0.01 vs control, **P < 0.01 vs PTZ and ^△^*P* < 0.05 or ^△△^*P* < 0.01 vs PTZ + SN. (PDF 142 kb)

Additional file 3: **Figure S3.** SN washout reverses its effect on the expression of hippocampal NLRP1 inflammasome complexes in PTZ kindled rats. Representative immunoreactive bands and statistical results showing SN washout reversed its effect on the expression of NLRP1 (A), ASC (B), and caspase-1 (C) in protein level. Data are expressed as means ± SEM. *n* = 6, ^##^*P* < 0.01 vs control, **P < 0.01 vs PTZ and ^△^*P* < 0.05 or ^△△^*P* < 0.01 vs PTZ + SN. (PDF 218 kb)

Additional file 4: **Figure S4.** SN washout reverses its effect on the expression of hippocampal inflammatory cytokines in PTZ kindled rats. Representative immunoreactive bands and statistical results showing SN washout reversed its effect on the expression of IL-1β (A), IL-18 (B), IL-6 (C), and TNF-α (D) in protein level. Data are expressed as means ± SEM. *n* = 6, ^##^*P* < 0.01 vs control, *P< 0.05 or **P < 0.01 vs PTZ and ^△^*P* < 0.05 or ^△△^*P* < 0.01 vs PTZ + SN. (PDF 287 kb)

## Abbreviations

AEDs: Antiepileptic drugs
ASC: Apoptosis-associated speck-like protein containing a caspase-activating recruitment domain
CNS: Central nervous system
ELISA: Enzyme-linked immunosorbent assay
H.E.: Hematoxylin and eosin
MWM: Morris water maze
NLRP1: Nucleotide oligomerization domain (NOD)-like receptor protein 1
TLE: Temporal lobe epilepsy
