# Supplementary figures and images for: Sinomenine exerts anticonvulsant profile and neuroprotective activity in pentylenetetrazole kindled rats: involvement of inhibition of NLRP1 inflammasome
Source: J Neuroinflammation. 2018 May 18;15:152. doi: 10.1186/s12974-018-1199-0 (PMC5960124; doi:10.1186/s12974-018-1199-0)

**Figure S1**

**A**

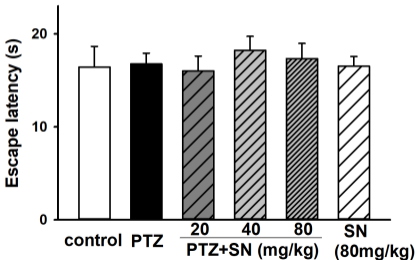

**B**

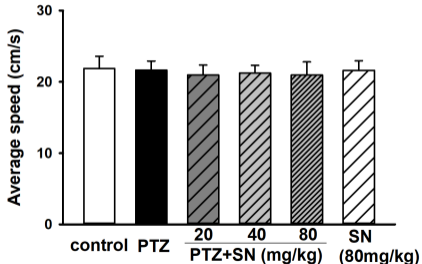

Supplement: Supplementary file 1 — Figure S1. The effect of sensorimotor ability and motivation on the escape latency and swimming speed. (A) and (B) Statistical results showing there were no differences in the escape latency and swimming speed among all groups in visible platform test. Data are expressed as means ± SEM. n = 10–12, P > 0.05. (PDF 71 kb) [file 12974_2018_1199_MOESM1_ESM.pdf]

**Figure S2**

**A**

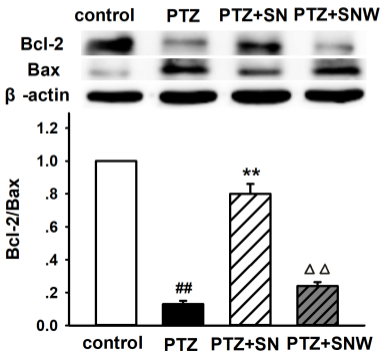

**B**

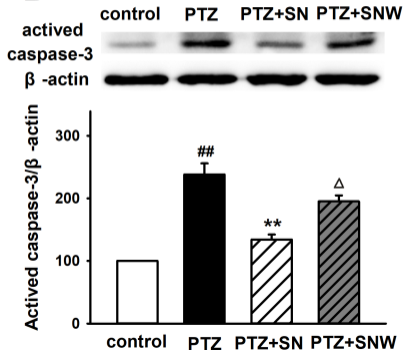

Supplement: Supplementary file 2 — Figure S2. SN washout reverses its effect on hippocampal apoptosis-related proteins in PTZ kindled rats. (A) Representative immunoreactive bands and statistical results showing SN washout reversed its effect on the ratio of Bcl-2/Bax. (B) Representative immunoreactive bands and statistical results showing SN washout reversed its effect on the expression of activated caspase-3. Data are expressed as means ± SEM. n = 6, ##P < 0.01 vs control, **P < 0.01 vs PTZ and △P < 0.05 or △△P < 0.01 vs PTZ + SN. (PDF 142 kb) [file 12974_2018_1199_MOESM2_ESM.pdf]

**Figure S3**

**A**

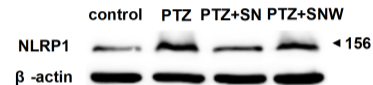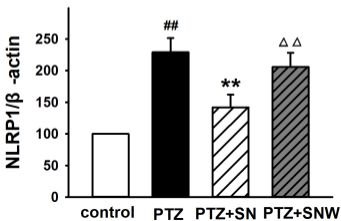

**B**

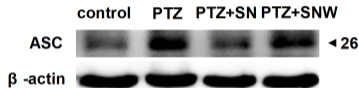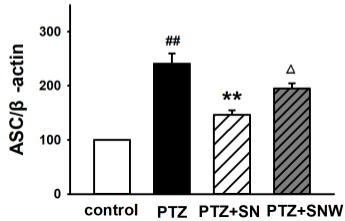

**C**

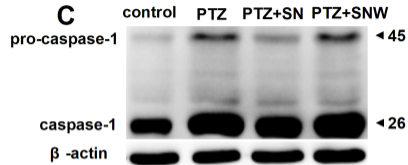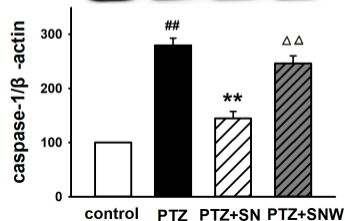

Supplement: Supplementary file 3 — Figure S3. SN washout reverses its effect on the expression of hippocampal NLRP1 inflammasome complexes in PTZ kindled rats. Representative immunoreactive bands and statistical results showing SN washout reversed its effect on the expression of NLRP1 (A), ASC (B), and caspase-1 (C) in protein level. Data are expressed as means ± SEM. n = 6, ##P < 0.01 vs control, **P < 0.01 vs PTZ and △P < 0.05 or △△P < 0.01 vs PTZ + SN. (PDF 218 kb) [file 12974_2018_1199_MOESM3_ESM.pdf]

**Figure S4**

**A**

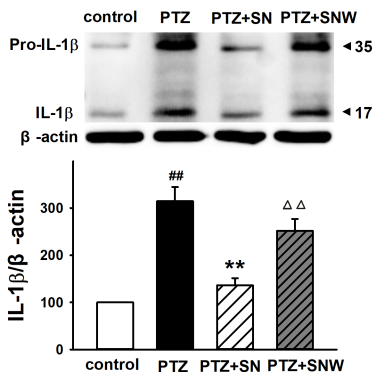

**B**

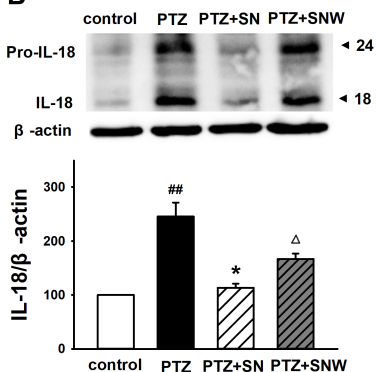

**C**

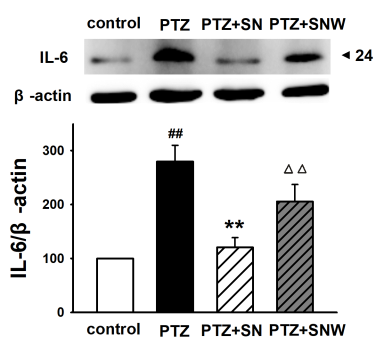

**D**

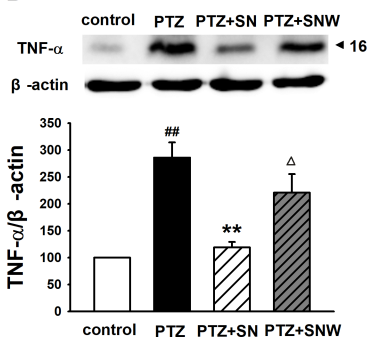

Supplement: Supplementary file 4 — Figure S4. SN washout reverses its effect on the expression of hippocampal inflammatory cytokines in PTZ kindled rats. Representative immunoreactive bands and statistical results showing SN washout reversed its effect on the expression of IL-1β (A), IL-18 (B), IL-6 (C), and TNF-α (D) in protein level. Data are expressed as means ± SEM. n = 6, ##P < 0.01 vs control, *P< 0.05 or **P < 0.01 vs PTZ and △P < 0.05 or △△P < 0.01 vs PTZ + SN. (PDF 287 kb) [file 12974_2018_1199_MOESM4_ESM.pdf]
